# Adverse Drug Reactions in Hospitalised Children in Germany Are Decreasing: Results of a Nine Year Cohort-Based Comparison

**DOI:** 10.1371/journal.pone.0044349

**Published:** 2012-09-18

**Authors:** Ann-Kathrin Oehme, Asia N. Rashed, Barbara Hefele, Ian C. K. Wong, Wolfgang Rascher, Antje Neubert

**Affiliations:** 1 Department of Paediatric and Adolescents Medicine, Friedrich-Alexander-University Erlangen/Nuremberg, Erlangen, Germany; 2 Centre for Paediatric Pharmacy Research, UCL School of Pharmacy, London, United Kingdom; 3 Department of Children and Adolescents Medicine Friedrich-Alexander-University Erlangen/Nuremberg, Erlangen, Germany; 4 Paediatric Clinical Study Center, Department of Paediatric and Adolescents Medicine Friedrich-Alexander-University Erlangen/Nuremberg, Erlangen, Germany; UCL Institute of Child Health, University College London, United Kingdom

## Abstract

**Background:**

In recent years, efforts have been made to improve paediatric drug therapy. The aim of this research was to investigate any changes regarding the frequency and nature of adverse drug reactions (ADRs) in hospitalized children in one paediatric general medical ward over a 9-year period.

**Methodology:**

Two prospective observational cohort studies were conducted at a large University hospital in Germany in 1999 and 2008, respectively. Children aged 0–18 years admitted to the study ward during the study periods were included. ADRs were identified using intensive chart review. Uni- and multivariable regression has been used for data analysis.

**Results:**

A total of 520 patients (574 admissions) were included [1999: n = 144 (167); 2008: n = 376 (407)]. Patients received a total of 2053 drugs [median 3, interquartile range (IQR) 2–5]. 19% of patients did not receive any medication. Median length of stay was 4 days (IQR 3–7; range 1–190 days) with a significantly longer length of stay in 1999. The overall ADR incidence was 13.1% (95% CI, 9.8–16.3) varying significantly between the two study cohorts [1999: 21.9%, 95% CI, 14.7–29.0; 2008: 9.2%, 95% CI, 5.9–12.5 (p<0.001)]. Antibacterials and corticosteroids for systemic use caused most of the ADRs in both cohorts (1999; 2008). Exposure to systemic antibacterials decreased from 62.9% to 43.5% whereas exposure to analgesics and anti-inflammatory drugs increased from 17.4% to 45.2%, respectively. The use of high risk drugs decreased from 75% to 62.2%. In 1999, 45.7% and in 2008 96.2% of ADRs were identified by treating clinicians (p<0.001).

**Conclusions:**

Between 1999 and 2008, the incidence of ADRs decreased significantly. Improved treatment strategies and an increased awareness of ADRs by physicians are most likely to be the cause for this positive development. Nevertheless further research on ADRs particularly in primary care and the establishment of prospective pharmacovigilance systems are still needed.

## Introduction

Over the last ten years the needs of children receiving pharmacotherapy have been increasingly recognized. Legislation was introduced in both the US [1] and, more recently, the EU [2] to facilitate the conduct of clinical trials in the paediatric population. Furthermore, funding was made available to establish paediatric networks and to increase research capacities [3].

Pharmacovigilance plays an important role in drug development and, because of the difficulties in conducting clinical trials, it is even more important in the paediatric population.

Investigating the frequency and nature of ADRs in children and adolescents is one important aspect of pharmacovigilance. Within the last few years various observational studies and meta-analyses were conducted to establish the epidemiology of ADRs in hospitalised children [4]–[11].

It has been shown that the incidence of ADRs in hospitalized children is about 10% [5], [6], [8],[12]. A large systematic review by Smyth et al indicated that the incidence rate for ADRs causing hospital admission is 2.9% [11]. An analysis of population based data revealed that about 2% of children taking medicines in the community experience an ADR [13].

At the Department of Paediatric and Adolescent Medicine, University Hospital Erlangen we conducted our first study investigating ADRs in children in 1999 [14]. Almost ten years later, in 2008 and within the set up of a larger international study (ADVISE) we collected similar data from the same ward [15]. ADVISE (Adverse Drug Reaction in Children – International Surveillance and Evaluation) is a multicentre study which investigated the incidence of ADRs in hospitalised children in five European and non-European countries.

Between the conduct of these two studies falls the introduction of diagnosis related groups (DRGs) for reimbursement of costs during hospitalization in our hospital. Contrary to previous methods this system is a case-based system, reimbursing hospitals for the treatment of patients based on the diagnosis and the procedures performed but independent of the length of hospital stay [16]. A reduction of the duration of hospital treatments was anticipated [17]. However, whether there is an impact on patients' safety so far remains unclear.

In the present manuscript we compare the results of these two cohort studies and investigate any changes in the frequency and nature of ADRs at our University Children Hospital between 1999 and 2008.

## Methods

### Study design

Two prospective observational cohort studies were conducted at the Department of Paediatric and Adolescents Medicine at the University Hospital Erlangen in Germany during an 8-month period from July 1999 to March 2000 and during a 3-month period from October to December in 2008, respectively. The first cohort study (Weiß et al study, 1999 cohort) was a pilot ADR-surveillance [14], the second cohort study (German part of ADVISE study, 2008 cohort) was part of the international ADVISE-project [15].

### Study setting

A general paediatric ward with a main focus on the treatment of infectious diseases. Between 1999 and 2008 the number of beds increased from 10 to 24 on this ward. Both studies were approved by the Ethics Committee at the University Hospital Erlangen.

### Study population

#### Inclusion criteria

All children aged 0 to 18 years admitted to the study ward within the respective study periods.

#### Exclusion criteria

Children with a length of hospital stay less than 24 hours; children with a main diagnosis of neoplasm.

### Database and data collection

Data collected in both cohort studies included: patient demographics (age, gender), medication data (dosage, route of administration, frequency, start and end date of each prescription) and admission diagnosis. For prescribed drugs each chemical compound or combination compound according to the Anatomic Therapeutic Chemical (**ATC**) classification was considered only once per patient [18]. Fluid and electrolyte infusions and parenteral nutrition were excluded.

Data were collected in a standardized format. For standardization the established international terminologies of **ATC** [18] for medication, International Classification of Diseases version 10 (**ICD10** [19]) for diagnosis and WHO Adverse Reaction Terminology (**WHO-ART**) [20] ) for adverse drug reactions were used.

Data from the 1999 cohort (Weiß et al study) were coded retrospectively for the purpose of this study and restricted to patients less than 18 years old. For the 2008 cohort (German part of ADVISE study) standardization was achieved through an online database application designed specifically for the ADVISE project (www.paediatric-adr.com).

### Adverse drug reactions (ADRs) identification

Adverse drug reactions were identified by intensive chart review, which is recognized as the gold standard for obtaining data on the incidence of ADRs [14], [21]. One researcher in each team screened all patient records regularly for events that could potentially be related to medication. This also included events which were present at admission and thus were potential ADRs leading to admission. All potential ADRs were then presented to the research team that reviewed the patient record including laboratory data and assessed whether the event was an ADR as defined by the WHO [22]. Each member of the team made his/her individual judgement and a final decision was made by consensus after discussion in the group. In both studies, ward staff was encouraged to monitor patients regarding ADRs.

### Assessment of ADRs

All ADRs were assessed using established algorithms. The causality/probability of ADRs was estimated using the Naranjo score [23] while severity was assessed using a weighted score published by Dormann et al. (2000) [24]. In regard to the preventability of an ADR the algorithm by Schumock and Thornton (1992) [25] was used. For each ADR the time of occurrence was documented as follows: , “before admission”, “before admission and reason for admission” and “during admission”.

### High risk drugs

Based on drug groups being described as most frequently involved in the occurrence of ADRs in the literature [26]–[30] and the opinions of two paediatric clinical pharmacologists involved in the project (W.R., N.C.) we defined five drug groups (ATC therapeutic level) as high risk: these were analgesics (N02), antiepileptics (N03), antibacterials and antimycotics for systemic use (J01, J02), corticosteroids for systemic use (H02), and immunosuppressant agents (L04) [31].

Other drugs were grouped as low risk drugs.

### Physicians' awareness

Physicians' awareness towards ADRs was also monitored. If relevant chart notes, changes in drug regimen, additional laboratory tests or other diagnostic measures were found in the patient's chart, the ADR was assumed to be “recognised”. If no evidence was found in the patient record that the responsible physician recognised the ADR, it was categorised as “not recognised”. Physicians' awareness was defined as the number of ADRs recognised by ward physicians divided by the total number of ADR.

### Statistical Analysis

Statistical analysis was performed using PASW Statistics 18 and Stata 11. Chi-squared-test was used to compare the proportions. In all statistical tests differences were considered to be significant at a p-value of <0.05.

Further analysis for possible risk factors that contribute to the occurrence of ADRs in each study cohort was conducted using univariable and multivariable regression methods. These analyses were conducted at patient level and ADR occurrence was used as the outcome measure. Initial association between individual variables and the incidence of ADRs was examined using a Chi-squared test. Univariable regression analysis was run for those variables which showed significance in the Chi-squared test. The multivariable logistic regression model included all variables that showed significant association in the univariable analysis. The full regression model was adjusted by length of stay and possible confounding factors (age, gender, numbers of low risk drugs and high risk drugs prescribed and diseases).

### Exposure rates

Overall exposure rates were defined as the number of patients receiving at least one prescription of a drug class divided by the total number of admissions during the study period. In addition we also calculated exposure rates for patients receiving at least one medication during their hospital stay. We calculated the exposure rates for analgesics, anti-inflammatory drugs and for antiinfectives for systemic use, because these were the most frequently prescribed drugs and the drug classes with the largest differences between the two studies.

### ADR incidence

The incidence of patients with ADRs was defined as the number of patients with an ADR divided by the number of patients receiving medications in the cohort.

In addition, we also calculated the proportion of patients experiencing an ADR. It was defined as the number of patients with at least one ADR divided by the total number of patients in the cohort and thus also including those patients not receiving any medication.

The proportion of all admissions that resulted from a drug taken prior to admission was calculated using the number of patients admitted due to an ADR divided by the total number of patients in the study cohort.

All results were multiplied by 100 and stratified by year of the study. For the purpose of incidence/proportion calculations and number of ADRs per patient, only the first patient admission was considered.

In order to adjust for the differences between the two cohort studies in observation periods and the duration of stay in hospital we also calculated the number of ADRs per 100 admissions, and per 100 days of hospital stay.

The total number of ADRs that occurred in the study cohort was based on all admissions.

## Results

### Study population

A total of 520 children (544 admissions) were included in the analysis [(1999 cohort): n = 144, (167); (2008 cohort): n = 376, (407)]. 229 children (44%) were female and the median age was 4 years (IQR 1–10, range 0–18 years). The total length of hospital stay in the whole population was 3231 days with a median of 4 days (IQR 3–7, range 0–190 days). Of the 520 hospitalised children 421 (81%) received at least one drug during their stay. In total 2053 drugs were prescribed to these 421 patients with a median of 3 drugs per patient (IQR 2–5, range 1–25). Demographic characteristics of children included from each study are shown in table 1. The length of hospitalisation decreased significantly from median 5 days to median 4 days (p<0.001) There was also a significant difference between the two studies with respect to age (p<0.05), number of diagnoses, number of patients on medication and number of drugs prescribed per patient (p<0.001). There was no significant difference in gender between the two study groups (p = 0.69) (Table 1).

**Table 1:** Comparison of patients' characteristics from two study cohorts.^†^

Patients characteristics		1999 Cohort^†^	95% CI	2008 Cohort^†^	95% CI	p-value*
**Number of patients (admissions)**		144 (167)	-	376 (407)	-	-
**Number of patients by age groups, No. (%):**	**0–≤2 years**	68 (47.2%)	38.6–55.6	133 (35.4%)	30.5–40.4	p<0.05
**Number of patients by age groups, No. (%):**	**>2–≤11 years**	50 (34.7%)	27.0–43.1	156 (41.5%)	36.5–46.7	p<0.05
	**>11–≤18 years**	26 (18.1%)	12.1–25.3	87 (23.1%)	19.0–27.7	p<0.05
**Age, years: median (IQR)** ††		3.0 (1–8.5)	2.0–4.0	5.0 (1–10.5)	4.0–6.0	-
**Gender, No. (%):**	**female** *	65 (45.8%)^1^	37.4–54.3	164 (43.6%)	38.5–48.8	p>0.05
	**male** *	77 (54.2%)^1^	45.7–62.6	212 (56.4%)	51.2–61.5	p>0.05
**Length of stay, days: median (IQR)** ††		5 (2–10)	4.0–6.0	4 (3–6)	3.0–4.0	p<0.001
**Diagnosis, no.: median (IQR)** ††		1.0 (1–2)	1.0–2.0	2.0 (1–3)	2.0–2.0	p<0.001
**Number of patients received medication (%)**		128 (88.9%)	82.6–93.5	293 (77.9%)	73.4–82.0	p<0.001
**Total number of drugs prescribed**		710	-	1343	-	-
**Number of drugs prescribed per patient in groups, No. (%):**	**no prescription**	16 (11.1%)	6.5–17.4	83 (22.1%)	18.0–26.6	p<0.001
	**1–<5 prescriptions**	78 (54.2%)	45.7–62.5	212 (56.4%)	51.2–61.5	p<0.001
	**5–10 prescriptions**	48 (33.3%)	25.7–41.7	64 (17.0%)	13.4–21.2	p<0.001
	**>10 prescriptions**	2 (1.4%)	0.2–4.9	17 (4.5%)	2.7–7.1	p<0.001
**Number of drugs prescribed per patient: median (IQR)** ††		3.0 (1.5–6)	2.0–4.0	2.0 (1–4)	2.0–3.0	p<0.01
**Number of drugs prescribed per patient (only those with medication): median (IQR)** ††		3.0 (2–6)	3.0–4.0	3.0 (2–5)	3.0–3.0	p<0.05
**Number of patients with high risk drugs (%)**		108 (75%/84.4%*)	67.1–81.8 76.9–90.2	234 (62.2%/79.8%*)	57.1–67.2 74.8–84.3	p<0.01 p>0.05

* Chi-Squared test;

1 two missings,

†† IQR = interquartile range.

### Diagnoses

In both studies the most common main diagnoses were infectious and parasitic diseases followed by respiratory system diseases.

**Figure 1:**
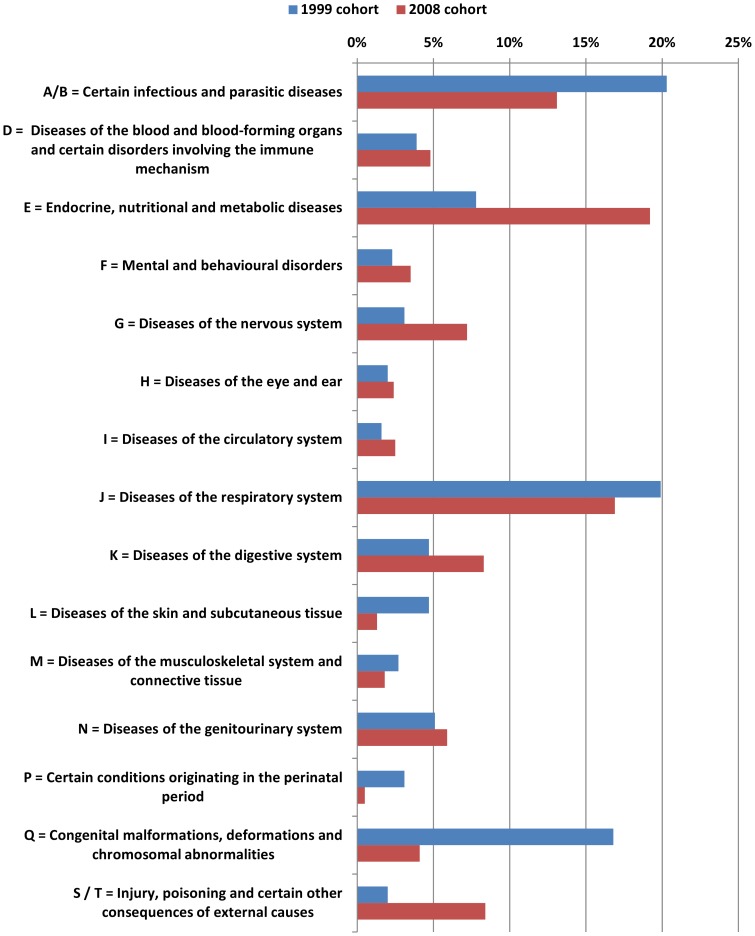
Main classes of diagnosis based on ICD10 for the two study cohorts.

In the 2008 cohort children with cystic fibrosis were less common but more children were admitted for monitoring purposes. Furthermore there was a higher number of diagnosis of ICD-class “E = endocrine, nutritional and metabolic diseases”. (Figure 1) The percentage of patients with more than 3 diagnoses was significantly higher in the 2008 cohort (17.3% vs. 5.6%, p<0.001). (Figure 1)

### Drug prescriptions

A total of 2053 prescriptions were documented in the study population. 710 prescriptions were recorded for the 1999 cohort and 1343 for the 2008 cohort, respectively.

In both study cohorts “antiinfectives for systemic use” were prescribed most frequently. Within this group the use of the subgroups “antibacterials for systemic use” (28.4% vs. 21.7%), “antimycobacterials” (1.7% vs. 0.1%) and “antimycotics for systemic use” (0.8% vs. 0.2%) decreased between 1999 and 2008 whereas the use of “antivirals for systemic use” (0.1% vs. 1.6%) increased.

**Figure 2:**
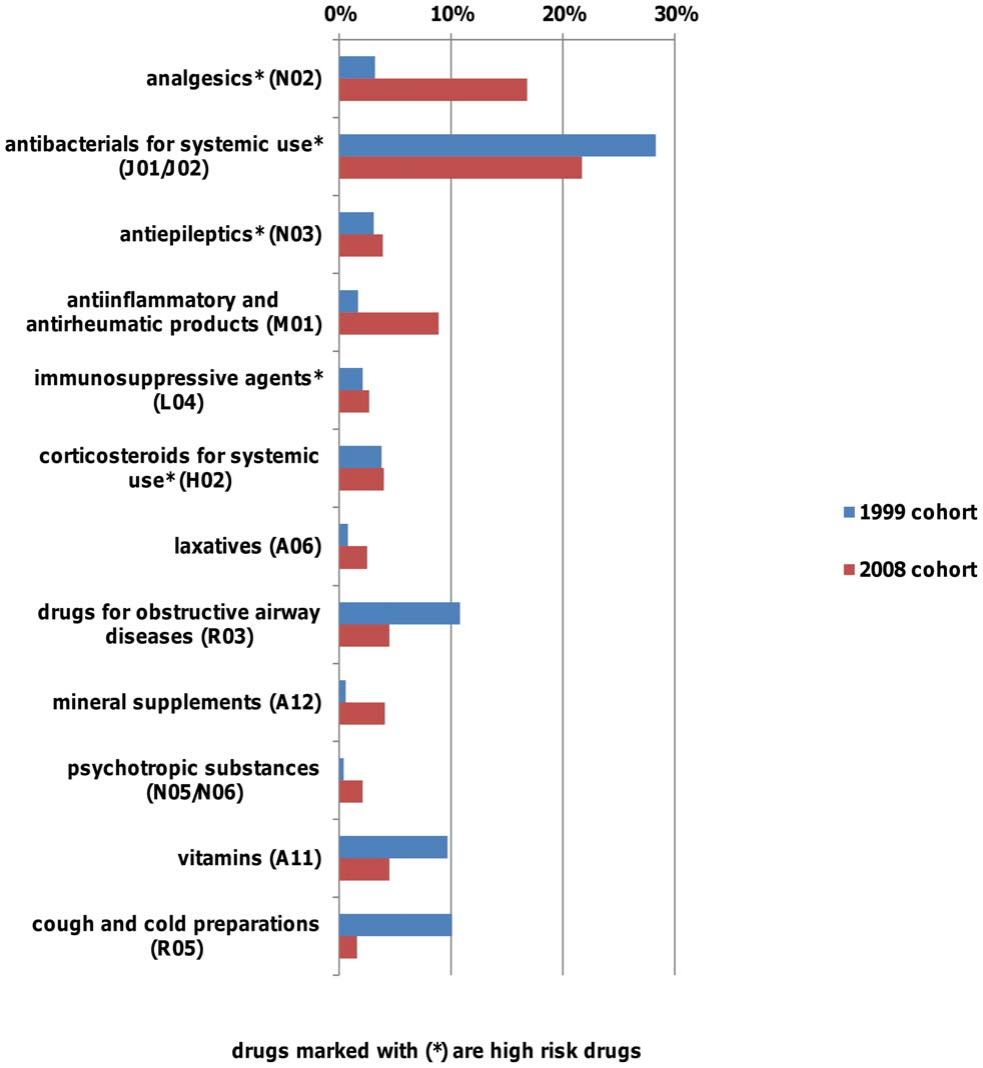
Most common prescribed drug classes in each study by ATC-T Level.

In the 1999 cohort, drugs for obstructive airway diseases (10.8%) and cough and cold medicines (10.1%) were the second most frequently prescribed, whereas in the 2008 cohort analgesics (16.8%) and anti-inflammatory & antirheumatic drugs (8.9%) were the second most frequently prescribed. The use of analgesics and anti-inflammatory drugs increased significantly from 3.3% in the 1999 cohort to 25.6% of all prescriptions in the 2008 cohort, respectively. (Figure 2)

### Exposure rates

Overall exposure rates, i.e. the number of children receiving at least one antiinfective for systemic use decreased from 62.9% to 42.5%. (Table 2) In contrast, the overall exposure rate for analgesics and anti-inflammatory drugs increased from 17.4% to 45.2%. Amongst patients receiving at least one medication the exposure rates changed from 82.0% to 59.0% for antiinfectives for systemic use and from 22.7% to 62.8% for analgesics and anti-inflammatory drugs, respectively. The drugs mostly responsible for this increase were metamizol, paracetamol and ibuprofen, which were almost not used at all in the 1999 cohort but prescribed to almost one third of the patients in the 2008 cohort (paracetamol = 24.6%, metamizol = 28.3%, ibuprofen = 29.2%). (Table 2)

**Table 2:** Exposure rates for most common antibacterials for systemic use and analgesics and anti-inflammatory drugs in two study cohorts.^†^

	1999 Cohort^†^			2008 Cohort^†^		
	no patients exposed	% all patients (n = 167)	% patients receiving medication (n = 128)	no patients exposed	% all patients (n = 407)	% patients receiving medication (n = 293)
**Antibacterials for systemic use (J01/J02)**	105	**62.9**	**82.0**	173	**42.5**	**59.0**
β-Lactam, penicillins (J01C)	33	19.8	25.8	61	15.0	20.8
Other β-Lactam antibiotics (cephalophorins, carbapenems) (J01D)	67	40.1	52.3	114	28.0	38.9
Macrolids, lincosamids and streptogramins (J01F)	19	11.4	14.8	19	4.7	6.5
Sulfonamids and trimethoprim (J01E)	16	9.6	12.5	17	4.2	5.8
Antimycotics for systemic use (J02A-V)	6	3.6	4.7	3	0.7	1.0
Aminoglycosids (J01G)	22	13.2	17.2	23	5.7	7.8
**Analgesics (N02)**	18	**10.8**	**14.1**	155	**38.1**	**52.9**
Paracetamol (N02BE0)	13	7.8	10.2	100	24.6	34.1
Metamizole (N02BB0)	6	3.6	4.7	115	28.3	39.2
Acetylsalicylic acid (N02BA01)	3	1.8	2.3		0.0	0.0
Tramadol (N02AX0)	0	0	0.0	6	1.5	2.0
Tilidine (N02AX0)	0	0	0.0	1	0.2	0.3
Buprenorphine (N02AE0)	0	0.0	0.0	1	0.2	0.3
Piritramide (N02AC0)	0	0.0	0.0	2	0.5	0.7
**Anti-inflammatory drugs (M01)**	12	**7.2**	**9.4**	**119**	**29.2**	**40.6**
Ibuprofen (M01AE0)	7	4.2	5.5	119	29.2	40.6
Indometacin (M01AB0)	5	3.0	3.9	0	0.0	0.0
**Analgesics and anti-inflammatory drugs**	29	**17.4**	**22.7**	184	45.2	62.8

### High risk drugs

The percentage of children with at least one prescription of a high risk drug decreased from 75% (95% CI, 67.1–81.8) to 62.2% (95% CI, 57.1–67.2) between the two time periods. Amongst those receiving at least one medication this percentage changed from 84.4% (95% CI, 76.9–90.2) to 79.8% (95% CI, 74.8–84.3), respectively. In the 1999 cohort significantly more children received more than one high risk drug (p<0.05). (Table 1)

### ADR incidence

55 out of 520 patients developed a total of 99 ADRs. The overall proportion of ADRs in the study cohort was 10.6% (95% CI, 7.9–13.2).

ADR incidence in the 1999 cohort was 21.9% (95% CI, 14.7–29.0) decreasing to 9.2% (95% CI, 5.9–12.5) in the 2008 cohort. In both studies the ADR incidence was found to be higher in older children (>11–18 years) although this association was stronger in 2008.

The proportion of patients with an ADR as a reason for admission was 0.0% in 1999 and 0.8% in 2008 (95% CI, −0.2–2.3) when 3 patients were hospitalised because of an ADR. (Table 3) Incidences based on other denominators are given in table 3.

**Table 3:** Frequency of adverse drug reactions (ADR).

	1999 Cohort % (95%-CI)	2008 Cohort % (95%-CI)	p-value	Overall % (95%-CI)
**ADR proportion**	n = 28/144, 19.4 (13.0–26.0)	n = 27/376, 7.2 (4.6–9.8)	<0.001	n = 55/520, 10.6 (7.9–13.2)
**ADR incidence**	n = 28/128, 21.9 (14.7–29.0)	n = 27/293, 9.2 (5.9–12.5)	<0.001	n = 55/421, 13.1 (9.8–16.3)
**No of ADR/100 admissions**	27.5 (20.7–34.3)	13.0 (9.9–16.7)	<0.001	17.2 (14.2–20.6)
**No of ADR/100 days in hospital**	4.4 (3.2–5.6)	2.6 (2.0–3.4)	0.09	3.6 (2.5–3.7)
**Proportion of patients with an ADR as reason for admission**	0.0	n = 3/376, 0.8 (0.2–2.3)	NA*	n = 3/520, 0.6 (0.1–1.7)

* NA = not applicable.

### ADR characteristics

In the 1999 cohort gastrointestinal-system disorders (n = 10; 21.7%), skin & appendages disorders (n = 9; 19.6%) and disorders of the white cells & the reticulo-endothelial system (n = 9; 19.6%) were the most frequently documented ADRs according to the WHO system organ classes, whereas in the 2008 cohort disorders of the white cells & the reticulo-endothelial system (n = 16; 30.2%) followed by metabolic & nutritional disorders (n = 10; 18.9%) and disorders of the central or peripheral nervous system (n = 6; 11.3%) were the ADR-classes observed most commonly.

117 drugs were suspected as a possible cause for these 99 ADRs. In both study cohorts antibacterials for systemic use were the drugs most often associated with ADRs [1999 cohort: n = 24 (11.9% of all prescriptions for antibacterials); 2008 cohort: n = 20 (6.9% of all prescriptions for antibacterials)], followed by corticosteroids for systemic use [1999 cohort: n = 9 (33.3% of all prescriptions for corticosteroids), 2008 cohort: n = 15 (27.8% of all prescriptions for corticosteroids), respectively]. (Table 4)

**Table 4:** ADR causative drugs and examples of ADRs for each study by ATC-T-level categories.†

Drug groups (ATC)	ADR causative drugs (n)/total drugs in group (n) (%)#		Examples of ADRs (causative drug)	
	1999 Cohort, Total n = 710	2008 Cohort, Total n = 1343	1999 Cohort	2008 Cohort
analgesics* (N02)	N = 0/23 (0.0%), 95%CI NA	N = 2/225 (0.9%), 95%CI (0.1–3.2)	-	thrombocytopenia (metamizol), sedation aggravated (tramadol)
antibacterials for systemic use* (J01/J02)	N = 24/201 (11.9%), 95%CI (7.8–17.2)	N = 20/291 (6.9%), 95%CI (4.2–10.4)	diarrhea (amoxicillin, imipenem), exanthema (amoxicillin, vancomycin), eosinophilia (cefaclor, cefotiam)	eosinophilia (vancomycin, cefotaxim, tobramycin), exanthema (amoxicillin, benzylpenicillin), neutropenia (cefotaxim)
antiepileptics* (N03)	N = 1/22 (4.5%), 95%CI (0.1–2.3)	N = 8/53 (15.1%), 95%CI (6.7–27.6)	elevated liver enzymes (phenobarbital)	thrombocytopenia (valproic acid), leucopenia (valproic acid, topiramate)
anti-inflammatory and antirheumatic products (M01)	N = 3/12 (25%), 95%CI (5.5–5.7)	N = 0/119 (0.0%), 95%CI NA	nausea & vomiting (indometacin), GI-bleeding (ibuprofen)	-
immuno-suppressive agents* (L04)	N = 3/15 (20%), 95%CI (4.3–4.8)	N = 8/36 (22.2%), 95%CI (10.1–39.2)	diarrhea & hypertrichosis (ciclosporin), herpes zoster (azathioprine)	hyponatriaemia & hyperkaliaemia (tacrolimus), hypertrichosis (ciclosporin)
corticosteroid for systemic use* (H02)	N = 9/27 (33.3%), 95%CI (16.5–54.0)	N = 15/54 (27.8%), 95%CI (16.5–41.6)	hypokaliaemia (methylprednisolone), leucocytosis (methylprednisolone)	hyperglycaemia, hypertension, Cushing's syndrome (prednisone), leucocytosis (prednisolone),
drugs for obstructive airway diseases (R03)	N = 2/77 (2.6%), 95%CI (0.3–9.1)	N = 0/60 (0.0%), 95%CI NA††	eosinophilia (theophylline), tachycardia (epinephrine)	-

Drugs marked with (*) are high risk drugs;

# percentage of total prescriptions in this group causing an ADR.

† most often prescribed drugs (n>25),

†† NA = not applicable as it is one-sided, 97.5% CI.

In 2008 the majority of ADRs was classified as mild (n = 48, 90.6%). In contrast in 1999 54.3% (n = 25) of ADRs were found to be mild and 41.3% (n = 19) were considered as moderate, respectively. Only one ADR (from 2008 cohort) was found to be severe.

### Physicians' awareness

The percentage of ADRs being recognized by the treating physician was 45.7% in the 1999 cohort and rose to 96.2% in the 2008 cohort (p<0.001).

### Regression

In both studies univariable analysis showed a significant relationship between the number of high risk drugs and the occurrence of ADRs. In the 1999 cohort we additionally found a significant association between the number of low risk drugs and the occurrence of ADRs and between diagnoses of ICD-Class “Q” and the occurrence of ADRs. In the 2008 cohort there was also a significant association for the number of diagnoses per patient as well as for diagnoses of ICD-Class “D”. (Table 5)

**Table 5:** Risk factors for ADRs in the two study cohorts (results of the univariable regression analysis).^†^

	1999 Cohort^†^		2008 Cohort^†^	
	OR (95%CI)	p-value	OR (95%CI)	p-value
Age				
0–≤2 years	1.4 (0.5–3.6)	0.508	0.7 (0.2–2.0)	0.501
2–≤11 years	1.0 (reference)		1.0 (reference)	
>11–18 years	1.1 (0.3–3.7)	0.924	2.3 (0.9–5.8)	0.071
Gender (female vs. male)	1.3 (0.5–2.9)	0.569	0.8 (0.3–1.7)	0.512
Number of diagnoses per patient	1.3 (1.0–1.7)	0.076	1.3 (1.1–1.5)*	<0.01
Length of hospital stay >7days	7.9 (0.9–65.5)	0.057	0.9 (0.1–9.7)	0.951
Number of low risk drugs prescribed				
0	1.0 (reference)		1.0 (reference)	
<5	3.5 (0.4–28.7)	0.236	1.0 (0.3–3.1)	0.996
5–10	11.5 (1.3–102.7)*	0.028	2.1 (0.5–9.1)	0.327
>10	NA††		7.7 (1.0–60.2)	0.053
Number of high risk drug prescribed				
0	1.0 (reference)		1.0 (reference)	
1	1.5 (0.1–15.8)	0.716	0.8 (0.1–5.6)	0.797
2–3	8.3 (1.0–67.0)*	0.047	2.8 (0.6–13.3)	0.190
>3	26.6 (2.6–269.4)*	<0.01	10.9 (2.3–51.2)*	<0.01
Special ICD10 diagnosis groups				
Diagnosis with code “D”**	NA††		11.4 (2.7–48.6)*	<0.01
Diagnosis with code “J”***	0.2 (0.1–1.0)	0.057	NA††	
Diagnosis with code “Q”****	4.1 (1.1–15.5)*	0.035	NA††	

* statistically significant variables (p<0.05);

** D = Diseases of the blood, the blood-forming organs and certain immune deficiencies;

*** J = Respiratory diseases;

**** Q = Congenital malformations, deformations and chromosomal abnormalities.

†† NA = Not applicable.

In the multivariable regression analysis the association between the number of high risk drugs (n>3) and the occurrence of ADRs only remained significant in the 1999 cohort (OR 12.8; 95% CI, 1.1–148.8). In the 2008 cohort this association became slightly insignificant, although the OR still indicates that children with >3 high risk drugs have a higher risk for ADRs (OR: 5.4, 95% CI, 0.9–33.8). Diagnosis with a “D”-code also remained significant in the 2008 cohort, however, this diagnosis was not reported in 1999. (Table 6)

**Table 6:** Risk factors for ADRs in the two study cohorts (results of the multivariable regression analysis).† *

Risk factors	1999 Cohort†		2008 Cohort†	
	OR (95% CI)	p-value	OR (95% CI)	p-value
Age (year)				
0–≤2 y	1.5 (0.4–5.2)	0.507	0.5 (0.1–1.6)	0.244
>2 y–≤11 y	1.0 (reference)		1.0 (reference)	
>11 y–≤18 y	1.0 (0.2–4.3)	0.986	2.0 (0.7–5.8)	0.183
Gender (female vs. male)	1.2 (0.4–3.7)	0.716	0.7 (0.3–1.8)	0.490
Number of diagnoses per patient	1.2 (0.8–1.8)	0.408	1.1 (0.9–1.3)	0.402
Length of hospital stay >7 days	5.6 (0.5–67.4)	0.175	2.3 (0.8–6.7)	0.146
Number of low risk drugs prescribed				
0	1.0 (reference)		1.0 (reference)	
<5	2.0 (0.2–20.5)	0.589	0.7 (0.2–2.8)	0.673
5–10	5.2 (0.4–61.1)	0.188	0.3 (0.05–2.1)	0.222
>10	NA††	-	0.6 (0.1–7.3)	0.709
Number of high risk drug prescribed				
0	1.0 (reference)		(reference)	
1	1.5 (0.1–17.4)	0.727	0.5 (0.1–4.1)	0.524
2–3	6.7 (0.7–62.4)	0.097	1.7 (0.3–9.4)	0.544
>3	12.8 (1.1–148.8)	0.041	5.4 (0.9–33.8)	0.072
Special ICD10 diagnosis groups				
Diagnosed with D**	NA††	NA††	7.4 (1.4–40.1)	0.020
Diagnosed with Q***	1.5 (0.3–8.1)	0.637	NA††	-
Diagnosed with J****	0.6 (0.1–3.5)	0.546	NA††	-

* full models were adjusted by possible confounding factors (age, gender, number of diagnosis, length of stay, number of drugs prescribed, and diseases.

** D = Diseases of the blood, the blood-forming organs and certain immune deficiencies;

*** Q = Congenital malformations, deformations and chromosomal abnormalities;

**** J = Respiratory diseases.

† Risk factors are presented as adjusted odds rations (ORs) with 95% confidence intervals.

†† NA = Not applicable.

There was no significant association between the length of hospital stay and the occurrence of ADRs in the univariable analysis in both studies. In the model of the 2008 cohort the OR for a length of stay >7 days was lower compared to the model of the 1999 cohort (2.3 vs. 5.6, respectively). However, there was no significant association with ADRs occurrence, but a lot of interactions with other factors.

## Discussion

We present the results of two studies which were conducted almost ten years apart using a similar methodology. We found an overall ADR incidence of 13.1% (95% CI, 9.8–16.3) which is slightly above the findings published previously [5], [6], [8], [12]. However, it is still lower than the overall incidence observed in our large international multi-centre study [15].

Our main finding is that there was a significant decrease of ADR incidence between 1999 and 2008. There are various reasons which may have led to this result.

### Polypharmacy

Polypharmacy has been associated with increased ADR incidences and has previously been shown to be a risk factor [12], [27], [32]. In our study the median number of drugs decreased from three in 1999 to two in 2008 in the overall population but did not change amongst those patients receiving at least one medication. Thus the decrease in ADR incidence cannot be explained by a decreasing use of medicines in general.

However, the multivariable regression analysis revealed that the number of high risk drugs is the only independent risk factor for the development of an ADR.

We determined that in the group of patients receiving medication the percentage of patients receiving at least one high risk drug decreased from 84.4% in 1999 to 79.8% in 2008. In addition high risk drugs such as antibacterials for systemic use were prescribed less often (28.4% to 21.7% of all prescriptions) and the exposure rate, i.e. the percentage of patients receiving at least one systemic antibacterial, decreased from 82.0% in 1999 to 59.9% in 2008, respectively. Altogether, this may explain the significantly decreasing ADR incidence over time in our study.

### Change of treatment strategies

Furthermore pharmacological treatment strategies on the ward have changed during the nine years and therefore probably influenced the incidence of ADRs.

For example more and different types of analgesics and anti-inflammatory drugs were prescribed during the 2008 cohort, e.g. we observed an increasing number of prescriptions for ibuprofen. Ibuprofen became more popular in recent years since some studies have proven the superior efficacy of ibuprofen compared to paracetamol, when treating fever in children [33], [34]. Increasing use of ibuprofen was reported previously from other countries such as the UK and Italy [35]–[37].

Furthermore, metamizol and paracetamol were also prescribed more often in 2008 than in 1999. However, they were not significantly associated with ADRs which confirms that both – and particularly paracetamol – are long-established drugs which are well studied in children and safe and effective when used appropriately.

The increasing use of metamizol is an interesting development. Some years ago metamizol was withdrawn from the market in some countries or became a prescription only medicine in others because of the risk of agranulocytosis. However, this adverse reaction is very rare. On the other side metamizol has good antipyretic and analgesic properties and can be administered intravenously which allows a fast pain and fever relief. [38]. Therefore in some countries such as Germany it is well accepted and commonly used in both children and adults.

Overall this development shows that the need for an adequate treatment of pain and fever in children has been realised and is currently put into practice in our hospital.

A similar picture as for analgesics was seen for systemic antibacterials. The proportion of drugs involved in ADRs compared to the total number of prescriptions in this group declined from 11.9% to 6.9%. Drugs which often caused ADRs in 1999 such as imipenem, vancomycin and streptomycin were prescribed less often in 2008.

This change may reflect the decreasing number of cystic fibrosis patients treated on the study ward because treatment strategies changed and their care – including treatment with parenteral antibiotics – was moved from hospital to ambulant settings [39]–[41].

### Length of hospitalisation

As anticipated with the introduction of DRGs [16], [17], [42] the length of hospitalisation significantly decreased between 1999 and 2008. Particularly the number of patients staying longer than 7 days in hospital decreased from 34% to 15.2%, respectively.

However, the univariable regression analysis showed no significant association between length of hospitalisation (LOS) and ADR occurrence in both studies. In the multivariable analysis, the OR to have an ADR for patients staying ‘>7 days’ was higher in the 1999 cohort as compared to the 2008 cohort but there was still no significant association with ADRs occurrence in both cohorts. This result is most likely to be explained by interactions that have occurred with other factors such as the number of drugs prescribed. Overall, the decrease in ADR incidence cannot be explained by the decrease of LOS but rather with changes in treatment strategies.

Nevertheless, as some ADRs take time to develop, they may have been missed in the 2008 cohort as the patient – because of the shorter LOS – was already discharged at the time the ADR occurred. Therefore further research into ADRs in the ambulatory care sector is needed to establish whether this is the case or not. So far the real impact of the introduction of DRGs on the incidence of ADRs remains unclear.

### Study population

Although both studies were conducted on the same ward and the dedication of the ward as predominantly infectious ward did not change, some differences in the study population were observed which may also have contributed to the decrease of ADR frequency. In 1999 more younger children (0–<2 years) were treated (47.2% vs. 35.4% of study population). One can argue that younger children are more vulnerable and thus more prone to developing adverse reaction to medications which could have contributed to the lower ADR incidence in 2008. However, in a previous large epidemiological study on ADR risk-factors we could not identify age as independent risk factor towards ADRs [15]. Similarly an extensive systematic review on ADRs in children did also not identify a significant relationship between younger age and the occurrence of ADRs. (p = 0.21) [11]

Despite the fact that infectious and parasitic diseases and respiratory infections were most frequent, the percentage of patients presenting with one of the above diagnoses within the study cohort was lower in 2008 as compared to 1999. Congenital malformations, deformations and chromosomal abnormalities were less frequent in 2008 as compared to 1999. On the other hand, in 2008 more endocrine, nutritional and metabolic disease were treated on the ward as compared to 1999.

Thus, it appears that patients in the 1999 cohort had more chronic and severe illnesses and thus needed more and stronger medication. This change is mainly related to the fact that fewer patients with cystic fibrosis were treated on the study ward because of a change in their treatment strategies. (See also study strengths and limitations).

### Physician's awareness

A strong increase in the detection of ADRs by treating physicians, (45.7% in 1999 and 96.2% in 2008 respectively) was identified. This implies that the awareness of healthcare professionals towards ADRs has been increased. Although the detection rate documented in 2008 (96.2%) might be slightly overestimated there was a significant increase in the physicians' attention to ADRs (p<0.001), which shows a positive development of ward staffs' alertness towards ADRs. A wide range of detection rates, between 42.5% in Neubert et al (2006), 89% in Aagaard et al (2010) and 91.1% in Haffner et al (2005) is reported in literature [4], [7], [43].

### Type and severity of ADRs

The proportion and incidence of ADRs decreased over the nine year period. Additionally, in 2008 the proportion of ADRs being moderate or severe was lower compared to 1999. However, this finding may, in part, be due to subjectivity of the assessment scale and differences in the two research groups.

In a previous multi-center study the inter-rater analysis of the severity assessment was moderate (k = 0.55) which indicated that there is some subjectivity in the severity assessment scale use [15]. Therefore interpretation of this finding needs to be cautious.

Differences in the pattern of ADRs might be, in part, linked to the differences in drug usage, which in turn may have led to different types of ADRs reported. For example, ADRs concerning white cells and reticulo-endothelial system were reported in 2008 cohort, while in the 1999 cohort gastrointestinal and cutaneous ADRs were the most frequent. The latter are frequently associated with antibiotic use which was less common in the 2008 cohort [12], [30].

### Study strengths and limitations

To our knowledge this is the first study to compare results of two prospective observational cohort studies conducted in one large academic children hospital in Germany at two time points. Both studies used intensive chart review to detect ADRs. This method is established as the gold standard for ADR detection [14], [21].

For data from the ADVISE study this method was assessed by re-evaluating 10 randomly selected patients by a second reviewer. The inter-rater agreement for identifying patients with ADRs was found to be “almost-perfect” with k = 0.89 (95%CI, 0.75–1.0), which confirms the validity of the method used in this study [15].

However, there are also several limitations for this analysis.

Because of an increase in the number of beds on the study ward between 1999 and 2008 the total number of patients included in the 2008 cohort was higher than in 1999 (376 vs. 144). Therefore the 1999 cohort is statistically less powerful.

In addition there was a transition of patients treated on the ward. For instance fewer patients with cystic fibrosis were treated because of switching parenteral antibiotic treatment from hospital to home parenteral therapy. Furthermore patients with concussion were admitted who did not receive any medication. However, we accounted for these patients in our results as we also ran the analysis for patients receiving at least one medication. The decrease in ADR incidence was similar; hence the decreasing number of patients receiving a medication was not the reason for the declining ADR incidence.

In 1999 the documentation and analysis of data relied on hand-written comments and manual transfer from the patients charts only. For the purpose of this study, standardized ICD10- and ATC-Codes had to be added manually. In 2008 the diagnosis and drugs were electronically transferred to the database, which may have provided a more complete picture. Nevertheless, the ADR incidence decreased although more information had been available.

## Conclusion

Over a 9-year period the incidence of ADRs decreased significantly on a general paediatric ward. Clinicians were also shown to be more aware of ADRs. Overall less medication is used and a clear change in the prescribing behaviour has been noticed. However it remains unclear to which extent there was a shift of the problem into primary care because of a decreasing length of hospital stay. Therefore further research on ADRs in primary care is needed.

Overall, the need for pharmacovigilance in the paediatric population, at least in our hospital has been realised. The next step will now be to establish prospective pharmacovigilance systems to improve medication safety in paediatrics.

## References

[1] US-Congress (2002) Best Pharmaceuticals for Children Act [online]. Public Law 107–109, Congressional Record of 107th US-Congress, Vol 147 (2001). Available: http://www.gpo.gov/fdsys/pkg/CRECB-2001-pt18/html/CRECB-2001-pt18-Pg25074-2.htm. Accessed 2012 Aug 01.

[2] EC (2006) Regulation (EC) No 1901/2006 of the European Parliament and of the Council on medicinal products for paediatric use [online]. Official Journal of the European Union. Available: http://eur-lex.europa.eu/LexUriServ/LexUriServ.do?uri=OJ:L:2006:378:0001:0019:en:PDF Accessed 2012 Aug 01.

[3] Ceci A, Giaquinto C, Aboulker JP, Baiardi P, Bonifazi F, et al (2009) The Task-force in Europe for Drug Development for the Young (TEDDY) Network of Excellence. Paediatr Drugs 11: 18–21. 19127946 10.2165/0148581-200911010-00008

[4] Aagaard L, Christensen A, Hansen EH (2010) Information about adverse drug reactions reported in children: a qualitative review of empirical studies. Br J Clin Pharmacol 70: 481–491. 20840440 10.1111/j.1365-2125.2010.03682.x PMC2950983

[5] Clavenna A, Bonati M (2009) Adverse drug reactions in childhood: a review of prospective studies and safety alerts. Arch Dis Child 94: 724–728. 19531524 10.1136/adc.2008.154377

[6] Gonzalez-Martin G, Caroca CM, Paris E (1998) Adverse drug reactions (ADRs) in hospitalized pediatric patients. A prospective study. Int J Clin Pharmacol Ther 36: 530–533. 9799056

[7] Haffner S, von Laue N, Wirth S, Thurmann PA (2005) Detecting adverse drug reactions on paediatric wards: intensified surveillance versus computerised screening of laboratory values. Drug Saf 28: 453–464. 15853446 10.2165/00002018-200528050-00008

[8] Impicciatore P, Choonara I, Clarkson A, Provasi D, Pandolfini C, et al (2001) Incidence of adverse drug reactions in paediatric in/out-patients: a systematic review and meta-analysis of prospective studies. Br J Clin Pharmacol 52: 77–83. 11453893 10.1046/j.0306-5251.2001.01407.x PMC2014499

[9] Lazarou J, Pomeranz BH, Corey PN (1998) Incidence of adverse drug reactions in hospitalized patients: a meta-analysis of prospective studies. JAMA 279: 1200–1205. 9555760 10.1001/jama.279.15.1200

[10] Martinez-Mir I, Garcia-Lopez M, Palop V, Ferrer JM, Rubio E, et al (1999) A prospective study of adverse drug reactions in hospitalized children. Br J Clin Pharmacol 47: 681–688. 10383547 10.1046/j.1365-2125.1999.00943.x PMC2014265

[11] Smyth RM, Gargon E, Kirkham J, Cresswell L, Golder S, et al (2012) Adverse drug reactions in children–a systematic review. PLoS One 7: e24061. 22403604 10.1371/journal.pone.0024061 PMC3293884

[12] Turner S, Nunn AJ, Fielding K, Choonara I (1999) Adverse drug reactions to unlicensed and off-label drugs on paediatric wards: a prospective study. Acta Paediatr 88: 965–968. 10519338 10.1080/08035259950168469

[13] Knopf H, Du Y (2010) Perceived adverse drug reactions among non-institutionalized children and adolescents in Germany. Br J Clin Pharmacol 70: 409–417. 20716242 10.1111/j.1365-2125.2010.03713.x PMC2949914

[14] Weiss J, Krebs S, Hoffmann C, Werner U, Neubert A, et al (2002) Survey of adverse drug reactions on a pediatric ward: a strategy for early and detailed detection. Pediatrics 110: 254–257. 12165575 10.1542/peds.110.2.254

[15] Rashed AN, Wong IC, Cranswick N, Hefele B, Tomlin S, et al (2012) Adverse Drug Reactions in Children–International Surveillance and Evaluation (ADVISE): a multicentre cohort study. Drug Saf 35: 481–494. 22612852 10.2165/11597920-000000000-00000

[16] Wilke MH, Hocherl E, Scherer J, Janke L (2001) [Introduction of the new DRG-based reimbursement system in German hospitals–a difficult operation? Experiences and possible solutions from the viewpoint of trauma surgery]. Unfallchirurg 104: 372–379. 11413951 10.1007/s001130050745

[17] Reinhold T, Thierfelder K, Muller-Riemenschneider F, Willich SN (2009) [Health economic effects after DRG-implementation–a systematic overview]. Gesundheitswesen 71: 306–312. 19288425 10.1055/s-0028-1119399

[18] WHO Collaborating Centre for Drug Statistics Methodology - Anatomic Therapeutic Chemical Classification index with DDDs [online]. Available: http://www.whocc.no/atc_ddd_index/ Accessed 2012 Aug 01.

[19] WHO (2011) International Classification of Diseases Version 10 [online]. Available: http://apps.who.int/classifications/icd10/browse/2010/en. Accessed 2012 Aug 01.

[20] WHO (2005) The WHO adverse reaction terminology: terminology for coding clinical information in relation to drug therapy [online]. Available: http://www.umc-products.com/graphics/3036.pdf. Accessed 2012 Aug 01. Genf.

[21] Buckley MS, Erstad BL, Kopp BJ, Theodorou AA, Priestley G (2007) Direct observation approach for detecting medication errors and adverse drug events in a pediatric intensive care unit. Pediatr Crit Care Med 8: 145–152. 17273111 10.1097/01.PCC.0000257038.39434.04

[22] WHO (1972) WHO technical report no. 498. International drug monitoring: the role of national centres [online]. Available: http://www.who-umc.org/graphics/24756.pdf Accessed 2012 Aug 01. WHO Meeting. Genf. 4625548

[23] Naranjo CA, Busto U, Sellers EM, Sandor P, Ruiz I, et al (1981) A method for estimating the probability of adverse drug reactions. Clin Pharmacol Ther 30: 239–245. 7249508 10.1038/clpt.1981.154

[24] Dormann H, Muth-Selbach U, Krebs S, Criegee-Rieck M, Tegeder I, et al (2000) Incidence and costs of adverse drug reactions during hospitalisation: computerised monitoring versus stimulated spontaneous reporting. Drug Saf 22: 161–168. 10672897 10.2165/00002018-200022020-00007

[25] Schumock GT, Thornton JP (1992) Focusing on the preventability of adverse drug reactions. Hosp Pharm 27: 538. 10118597

[26] Choonara I, Rieder MJ (2002) Drug Toxicity and Adverse Drug Reactions in Children - A Brief Historical Review. Paediatric and Perinatal Drug Therapy 5: 12–18.

[27] Pirmohamed M, James S, Meakin S, Green C, Scott AK, et al (2004) Adverse drug reactions as cause of admission to hospital: prospective analysis of 18 820 patients. BMJ 329: 15–19. 15231615 10.1136/bmj.329.7456.15 PMC443443

[28] Thuermann PA, Windecker R, Steffen J, Schaefer M, Tenter U, et al (2002) Detection of adverse drug reactions in a neurological department: comparison between intensified surveillance and a computer-assisted approach. Drug Saf 25: 713–724. 12167067 10.2165/00002018-200225100-00004

[29] van den Bemt PM, Egberts AC, Lenderink AW, Verzijl JM, Simons KA, et al (2000) Risk factors for the development of adverse drug events in hospitalized patients. Pharm World Sci 22: 62–66. 10849925 10.1023/a:1008721321016

[30] van der Hooft CS, Dieleman JP, Siemes C, Aarnoudse AJ, Verhamme KM, et al (2008) Adverse drug reaction-related hospitalisations: a population-based cohort study. Pharmacoepidemiol Drug Saf 17: 365–371. 18302300 10.1002/pds.1565

[31] Rashed AN, Wong IC, Cranswick N, Tomlin S, Rascher W, et al (2012) Risk factors associated with adverse drug reactions in hospitalised children: international multicentre study. Eur J Clin Pharmacol 68: 801–810. 22166934 10.1007/s00228-011-1183-4

[32] Zopf Y, Rabe C, Neubert A, Hahn EG, Dormann H (2008) Risk factors associated with adverse drug reactions following hospital admission: a prospective analysis of 907 patients in two German university hospitals. Drug Saf 31: 789–798. 18707193 10.2165/00002018-200831090-00007

[33] Hay AD, Redmond NM, Costelloe C, Montgomery AA, Fletcher M, et al (2009) Paracetamol and ibuprofen for the treatment of fever in children: the PITCH randomised controlled trial. Health Technol Assess 13: iii–iv, ix–x, 1–163. 10.3310/hta13270 19454182

[34] Perrott DA, Piira T, Goodenough B, Champion GD (2004) Efficacy and safety of acetaminophen vs ibuprofen for treating children's pain or fever: a meta-analysis. Arch Pediatr Adolesc Med 158: 521–526. 15184213 10.1001/archpedi.158.6.521

[35] Carrasco-Garrido P, Jimenez-Garcia R, Barrera VH, de Andres AL, de Miguel AG (2009) Medication consumption in the Spanish paediatric population: related factors and time trend, 1993–2003. Br J Clin Pharmacol 68: 455–461. 19740404 10.1111/j.1365-2125.2009.03449.x PMC2766486

[36] Neubert A, Verhamme K, Murray ML, Picelli G, Hsia Y, et al (2010) The prescribing of analgesics and non-steroidal anti-inflammatory drugs in paediatric primary care in the UK, Italy and the Netherlands. Pharmacol Res 62: 243–248. 20451614 10.1016/j.phrs.2010.04.006

[37] Sturkenboom MC, Verhamme KM, Nicolosi A, Murray ML, Neubert A, et al (2008) Drug use in children: cohort study in three European countries. BMJ 337: a2245. 19029175 10.1136/bmj.a2245 PMC2593449

[38] Schonhofer P, Offerhaus L, Herxheimer A (2003) Dipyrone and agranulocytosis: what is the risk? Lancet 361: 968–969. 12648993 10.1016/S0140-6736(03)12751-1

[39] Stern M, Wiedemann B, Wenzlaff P (2008) German Cystic Fibrosis Quality Assessment G (2008) From registry to quality management: the German Cystic Fibrosis Quality Assessment project 1995 2006. Eur Respir J 31: 29–35. 17898017 10.1183/09031936.00056507

[40] van Westreenen M, Tiddens HA (2010) New antimicrobial strategies in cystic fibrosis. Paediatr Drugs 12: 343–352. 21028914 10.2165/11316240-000000000-00000

[41] Wiedemann B, Steinkamp G, Sens B, Stern M (2001) German Cystic Fibrosis Quality Assurance G (2001) The German cystic fibrosis quality assurance project: clinical features in children and adults. Eur Respir J 17: 1187–1194. 11491163 10.1183/09031936.01.00053901

[42] Rogers WH, Draper D, Kahn KL, Keeler EB, Rubenstein LV, et al (1990) Quality of care before and after implementation of the DRG-based prospective payment system. A summary of effects. JAMA 264: 1989–1994. 2120478

[43] Neubert A, Dormann H, Weiss J, Criegee-Rieck M, Ackermann A, et al (2006) Are computerised monitoring systems of value to improve pharmacovigilance in paediatric patients? Eur J Clin Pharmacol 62: 959–965. 17021890 10.1007/s00228-006-0197-9

